# KDM4A as a prognostic marker of oral squamous cell carcinoma: Evidence from tissue microarray studies in a multicenter cohort

**DOI:** 10.18632/oncotarget.18302

**Published:** 2017-05-30

**Authors:** Xin Jin, Hao Xu, Xingyu Wu, Taiwen Li, Jing Li, Yu Zhou, Hongxia Dan, Lu Jiang, Xin Zeng, Ping Ji, Qianming Chen

**Affiliations:** ^1^ College of Stomatology, Chongqing Medical University, Chongqing, China; ^2^ State Key Laboratory of Oral Diseases, National Clinical Research Center for Oral Diseases, West China Hospital of Stomatology, Sichuan University, Chengdu, China; ^3^ Chongqing Key Laboratory of Oral Diseases and Biomedical Sciences, Chongqing, China; ^4^ Chongqing Municipal Key Laboratory of Oral Biomedical Engineering of Higher Education, Chongqing, China

**Keywords:** KDM4A, oral squamous cell carcinoma, prognosis, survival, tissue microarrays

## Abstract

**Purpose:**

Previous studies have identified histone demethylase KDM4A to be a key epigenetic priming factor for the invasive squamous cell carcinoma growth and metastasis. The purpose of this study was to examine KDM4A as an independent prognostic marker in oral squamous cell carcinoma, using multicenter tissue microarrays.

**Results:**

The expression of KDM4A was significantly correlated with lymph node metastasis and TNM stage. KDM4A overexpression was associated with poor overall survival, and it was found to be a statistically significant independent predictor of all-cause mortality. These findings are validated by external TCGA HNSCC data. Addition of KDM4A expression improved the discriminatory accuracy of standard clinicopathologic features for prediction of cancer-specific survival (Model 4, area under the curve = 0.740, 95% confidence interval = 0.685 to 0.795, and Model 3, AUC = 0.695, 95% CI = 0.637 to 0.753, respectively).

**Materials and Methods:**

KDM4A expression was measured by immunohistochemistry, using tissue microarrays of OSCC samples collected from 313 patients. Kruskal-Wallis and chi-square tests were applied to investigate the correlation between KDM4A expression and clinicopathological factors. Overall survival analysis was performed using the Kaplan-Meier and multivariable logistic regression models, and the predictive ability of KDM4A in combination with known OSCC risk factors was evaluated. Receiver operating characteristic curves were used to assess discriminatory accuracy of these models. Additionally, disease-free survival was analyzed in patients with head and neck SCC reported on The Cancer Genome Atlas database.

**Conclusions:**

KDM4A expression is an independent predictor for the survival time of patients with OSCC and may be a valuable consideration to postoperative treatment options.

## INTRODUCTION

Oral squamous cell carcinoma (OSCC) is one of the most common malignancies worldwide. Despite improvements in clinical management, it continues to have high local recurrence and poor 5-year survival rate [1, 2]. Local tumor recurrence occurs in approximately 60% of patients, and metastasis affects 15–25%, leading to a poor prognosis [3]. This demonstrates the need of further investigating the factors associated with disease outcome and the development of novel postoperative treatments. Therefore, the identification of prognostic predictors has become an important issue in the management of OSCC.

Cell function is often regulated by environmental susceptibility factors that trigger modifications in chromatin structure and regulate gene expression [4]. Those modifications, including covalent DNA methylation and non-covalent histone modifications are collectively known as epigenetic mechanisms. Lysine methylation is one of the most notable posttranslational modifications and its dysregulation is involved in cancer progression. KDM4A is the most studied enzyme that is capable of demethylating lysine residues, specifically lysine 9 and 36 on histone H3. Studies have focused on KDM4A activity and its contribution in transcription regulation, where it may either stimulate or repress gene transcription [5, 6].

According to a previous study, histone demethylase KDM4A is a key epigenetic factor that activates genes encoding the AP-1 transcription factor, which thereby promotes head and neck SCC invasive growth and metastasis. KDM4A protein was significantly increased in metastatic lymph nodes compared with that in primary human SCC, indicating that KDM4A has a critical role in promoting human SCC metastasis. This finding provides new insights into the epigenetic and molecular control of human SCC metastasis [7]. Although substantial progress has been made in understanding the epigenetic events of SCC invasion and metastasis, very little is known about the prognostic value of epigenetic regulation in patients with OSCC. Thus, histone demethyases have not yet been used in clinical settings.

At present, the tumor-node-metastasis (TNM) staging system is known as the most prognostic tool for tumor survival [8]. Clinical characteristics of patients are also important for therapeutic planning and complications risk determination. Recently, age [9], smoking habits [10], extracapsular spread (ECS) in the cervical lymph nodes [11], bony involvement [12], tumor size, and microvascular invasion [13] have been showed to be independent prognostic factors associated with OSCC patients. However, the predictive effect is poor, as the majority of findings have been derived from small samples of a single center. Although clinicopathological data, including clinical TNM stage, tumor cell differentiation, tumor size, and lymph node metastasis are related to the survival time, the poor prognosis also occurs among patients with high differentiation, early clinical stage, or without lymph node metastasis. In this aspect, prognostic factors cannot be accurately assessed in a “low-risk” population.

We aimed to verify the epigenetic activation of KDM4A in OSCC tissues from a larger sample size, using multicenter tissue microarrays (TMAs). We also identified whether increased tissue expression of KDM4A is associated with poor clinical outcome of OSCC patients, independently of known clinical risk factors. In this way, we improved the prediction value of OSCC prognosis beyond existing prediction models.

## RESULTS

### Study population

**Figure 1:**
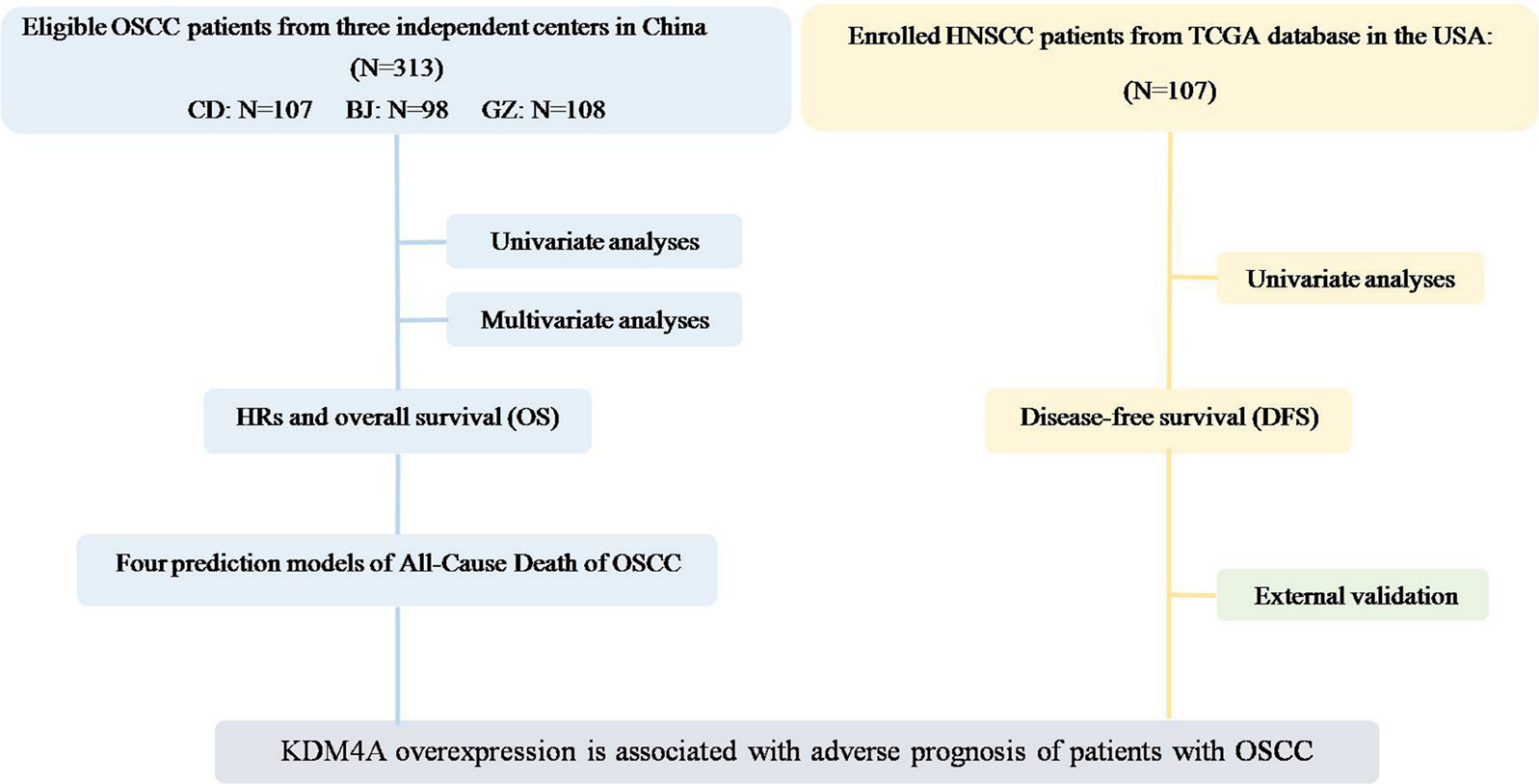
Study flow diagram

A total of 313 OSCC patients from three independent centers were participated in this cohort study (Figure 1). This study consisted of 169 men and 63 women with ages ranging from 24 to 83 years. To further examine the role of KDM4A in OSCC progression, related clinicopathological parameters were investigated through statistical analysis. The results showed that increased expression of KDM4A in OSCC was significantly associated with increased frequencies of lymph node metastasis (*P* = .017) and high clinical TNM stage (*P* = .011).The basic characteristics of the 313 study participants and distributions of KDM4A by selected study variables are presented in Table 1.

**Table 1:** Baseline characteristics of the patients with OSCC and distribution of KDM4A by selected study variables

Variable		No. of Patients No. (%)	KDM4A		*P* Value^#^
			Low expression No. (%)	High expression No. (%)	
Sex	Male	213 (68.05)	99 (46.48)	114 (53.52)	0.477
	Female	100 (31.95)	53 (53.00)	47 (47.00)	
Age	> 60 yr	154 (49.20)	84 (52.60)	73 (47.4)	0.260
	≤ 60 yr	159 (50.80)	71 (44.65)	88 (55.35)	
Smoking history	Never	167 (53.35)	80 (47.90)	87 (52.10)	0.903
	Ever	146 (46.65)	72 (49.32)	74 (50.68)	
Drinking history	Never	182 (58.15)	90 (49.45)	92 (50.55)	0.732
	Ever	131 (41.85)	62 (47.33)	69 (52.67)	
Location	Buccal mucosa	52 (16.61)	27 (51.92)	25 (48.08)	0.991
	Tongue	114 (36.42)	54 (47.37)	60 (52.63)	
	Gingiva	66 (21.09)	34 (51.52)	32 (48.48)	
	Others*	81 (25.88)	37 (45.68)	44 (54.32)	
Cell differentiation	High	193 (61.34)	96 (49.48)	97 (50.52)	0.951
	Moderate	92 (29.39)	42 (45.65)	50 (54.35)	
	Low	28 (8.95)	14 (50.00)	14 (50.00)	
Tumor stage	T1	45 (14.38)	21 (46.67)	24 (53.33)	0.101
	T2	139 (44.41)	71 (51.08)	68 (48.92)	
	T3	61 (19.49)	35 (57.38)	26 (42.62)	
	T4	68 (21.73)	25 (36.76)	43 (63.24)	
Lymph node metastasis	No	168 (53.67)	94 (55.95)	74 (44.05)	**0.017**
	Yes	145 (46.33)	68 (40.00)	87 (60.00)	
TNM stage	I	37 (11.82)	23 (62.16)	14 (37.84)	**0.011**
	II	94 (30.03)	55 (58.51)	39 (41.49)	
	III	127 (40.58)	55 (43.31)	72 (56.69)	
	IV	55 (17.57)	19 (34.55)	36 (65.45)	

Abbreviations: *P* Value^#^ was determined using the Kruskal-Wallis and chi-square tests.

Others* included floor of mouth, lips and palate.

### Survival analysis

**Figure 2:**
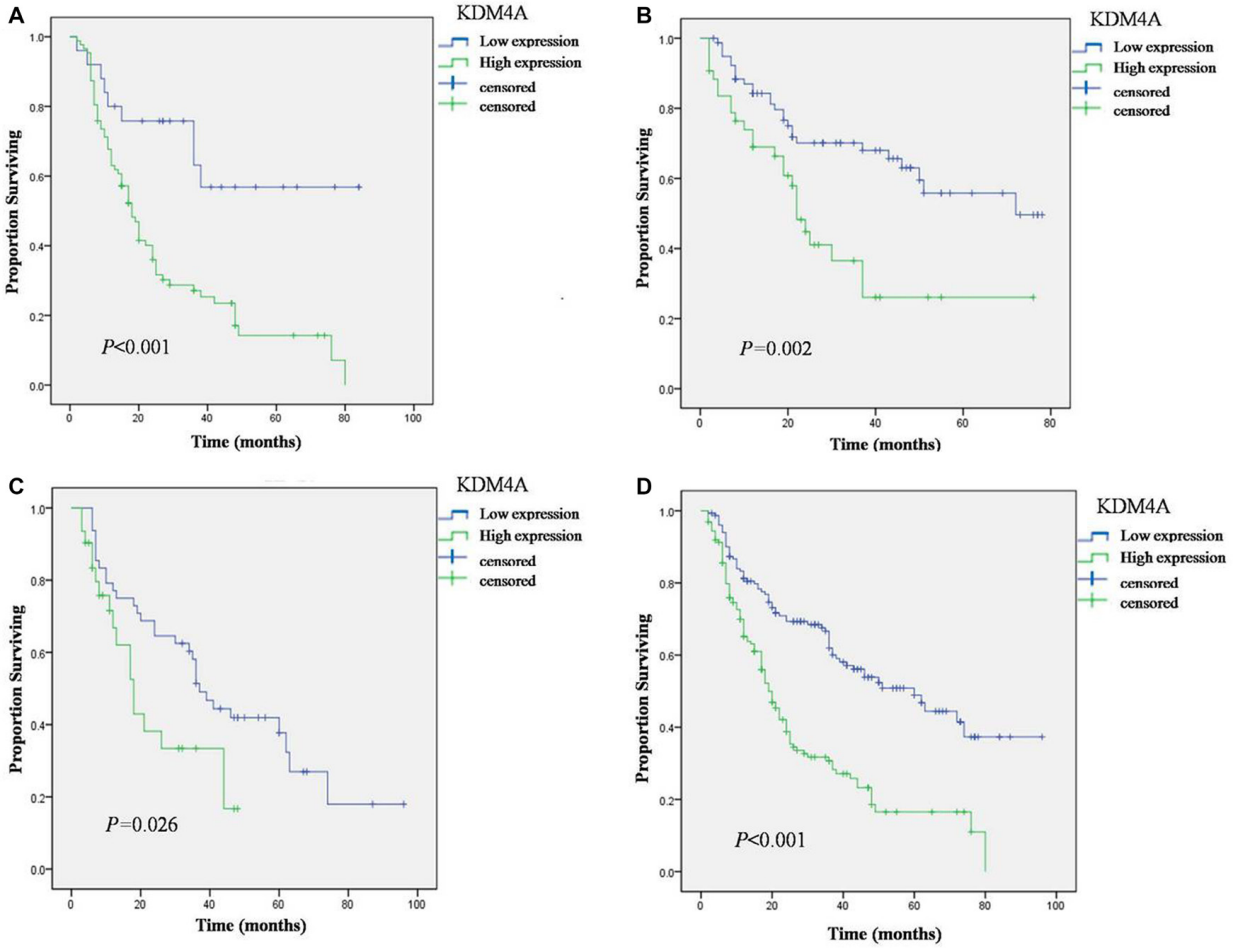
Overall Survival (OS) of OSCC patients with high and low expression of KDM4A in three cohorts defined by the Kaplan-Meier survival curves (**A**) Overall Survival in Chengdu cohort. (**B**) Overall Survival in Guangzhou cohort. (**C**) Overall Survival in Beijing cohort. (**D**) Overall Survival in joint cohort.

In Kaplan-Meier analysis, KDM4A expression, large tumor size, late clinical TNM stage, and positive lymph node metastasis were significant risk factors in patients who underwent OSCC resection (Table 2). For the Chengdu, Guangzhou, Beijing and joint cohorts, it is noteworthy that the strong staining group was significantly associated with low overall survival rate than the weak staining group (Figure 2A–2D). However, no significant correlation was observed between the overall survival and sex, age (< 60 or > 60), smoking, drinking, tumor site, and cell differentiation.

**Table 2:** Univariate analysis of selected characteristics and overall survival of patients with OSCC

Variable		*P* Value^#^
Sex	Male	0.524
	Female	
Age	> 60 yr	0.25
	≤ 60 yr	
Smoking history	Never	0.179
	Ever	
Drinking history	Never	0.07
	Ever	
Location	Buccal mucosa	0.713
	Tongue	
	Gingiva	
	Others*	
Cell differentiation	High	0.371
	Moderate	
	Low	
Tumor stage	T1 + T2	**0.049**
	T3 + T4	
Lymph node metastasis	No	**0.003**
	Yes	
TNM stage	I + II	**0.001**
	III + IV	
KDM4A	Low expression	**< 0.001**
	High expression	

Abbreviations: *P* Value^#^ was determined using the log-rank test.

Others* included floor of mouth, lips and palate.

**Figure 3:**
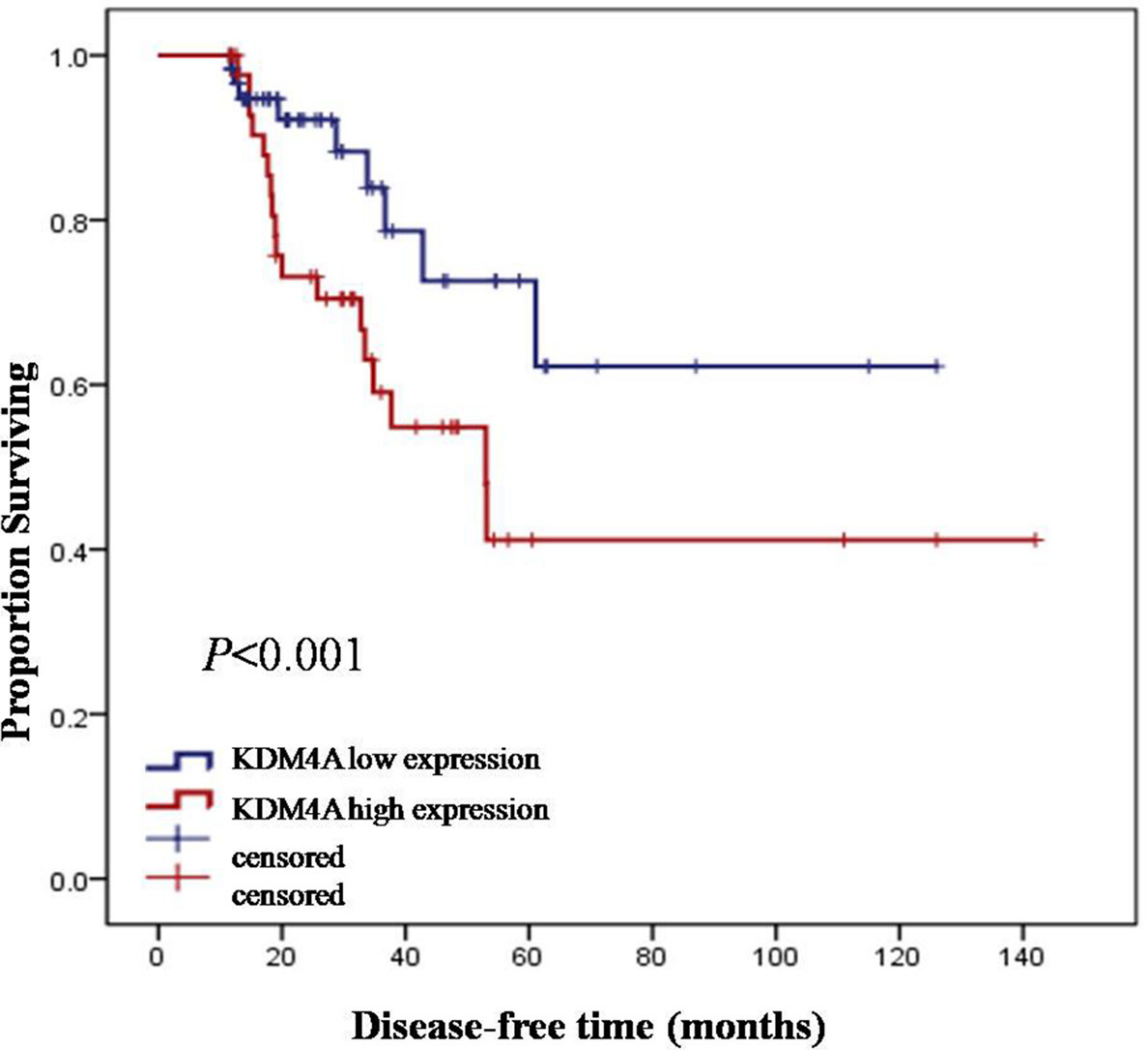
Kaplan-Meier estimates of disease-free survival (DFS) in relation to KDM4A expression

In the external validation cohort of 107 patients reported on TCGA database (2015), who had tumor recurrence records, the Kaplan-Meier estimates of disease-free survival (DFS) revealed that high KDM4A expression was significantly associated with the time of disease recurrence and progress (log-rank, *P* < .05), and consequently, with poor survival (Figure 3).

Multivariate Cox proportional hazards survival analysis was next employed to identify the prognostic value of KDM4A. We developed 4 risk prediction models. Risk predictors that were significantly associated with increased or decreased risks of OSCC in the multivariable models are shown in Table 3.

**Table 3:** Multivariate analysis of overall survival of patients with OSCC

Variable		Model 1	Model 2	Model 3	Model 4
		HR (95% CI)	HR (95% CI)	HR (95% CI)	HR (95% CI)
Sex	Male	1 ref	1 ref	1 ref	1 ref
	Female	0.819 (0.566,1.186)	0.874 (0.598,1.278)	0.950 (0.630,1.433)	0.966 (0.634,1.471)
Age	> 60 yr	1 ref	1 ref	1 ref	1 ref
	≤ 60 yr	1.029 (0.759,1.394)	1.039 (0.764,1.413)	0.980 (0.716,1.34)	0.987 (0.719,1.354)
Smoking history	Never	1 ref	1 ref	1 ref	1 ref
	Ever	0.885 (0.59,1.327)	0.943 (0.631,1.409)	0.784 (0.518,1.187)	0.876 (0.580,1.324)
Drinking history	Never	1 ref	1 ref	1 ref	1 ref
	Ever	0.909 (0.616,1.342)	0.876 (0.595,1.291)	0.949 (0.641,1.405)	0.910 (0.616,1.342)
Location	Buccal mucosa	1 ref	1 ref	1 ref	1 ref
	Tongue	1.564 (0.97,2.522)	1.569 (0.970,2.538)	1.615 (0.986,2.647)	**1.646 (1.002,2.703)**
	Gingiva	1.562 (0.935,2.609)	1.570 (0.939,2.625)	1.447 (0.840,2.491)	1.444 (0.834,2.498)
	Others*	**1.682 (1.035,2.735)**	**1.686 (1.035,2.749)**	**1.685 (1.022,2.779)**	**1.687 (1.026,2.775)**
Cell differentiation	High			1 ref	1 ref
	Moderate			0.833 (0.578,1.199)	0.887 (0.615,1.279)
	Low			1.189 (0.697,2.029)	1.272 (0.748,2.164)
Tumor stage	T1			1 ref	1 ref
	T2			0.768 (0.427,1.382)	0.794 (0.441,1.429)
	T3			0.767 (0.395,1.49)	0.872 (0.447,1.701)
	T4			1.15 (0.586,2.256)	1.204 (0.615,2.357)
Lymph node metastasis	No			1 ref	1 ref
	Yes			**1.907 (1.095,3.322)**	1.723 (0.833,3.565)
TNM stage	I			1 ref	1 ref
	II			1.929 (0.936,3.976)	1.723 (0.833,3.565)
	III			1.449 (0.621,3.377)	1.160 (0.491,2.743)
	IV			1.412 (0.545,3.658)	1.108 (0.425,2.890)
KDM4A	Low expression		1 ref		1 ref
	High expression		**2.426 (1.177,3.311)**		**2.191 (1.592,3.016)**

Abbreviations: HR, Hazard Ratio.

Others* included floor of mouth, lips and palate.

Tumor on the floor of the mouth, lips, or palate had decreased overall survival in all 4 models, with hazard ratios (HRs) of 1.682 (95% confidence interval [CI] = 1.035 to 2.735), 1.686 (95% CI = 1.035 to 2.749), 1.685(95% CI = 1.022 to 2.779) and 1.687 (95% CI = 1.026 to 2.775), respectively. In model 1 (clinical characteristics only), poor prognosis was significantly associated with tumor location. In model 2 (KDM4Aand clinical characteristics), increased KDM4A expression was more significantly associated with the risk of mortality, than clinical characteristics only (strong staining vs. weak staining, HR = 2.426, 95% CI = 1.177 to 3.311). In model 3 (clinical and pathological characteristics), lymph node metastasis was significantly associated with poor prognosis. Finally, in model 4 (extended by adding KDM4A), high KDM4A expression level was also associated with significantly high death risk (strong staining vs. weak staining, HR = 2.191, 95% CI = 1.592 to 3.016). These results suggested that KDM4A is an independent prognostic marker.

### Prediction models

**Figure 4:**
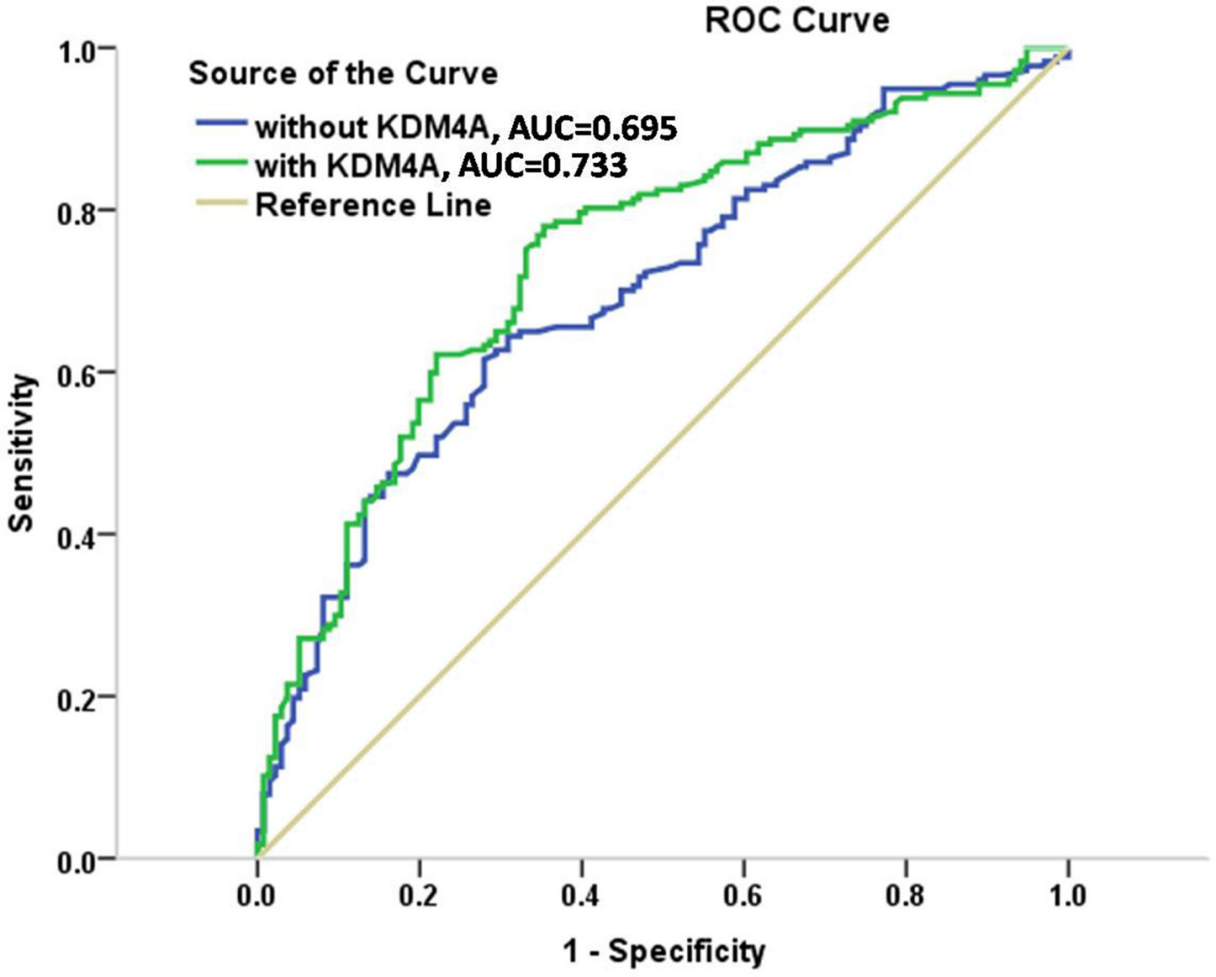
ROC curves for models with and without KDM4A expression in the cohorts

The improvement in discrimination was assessed by comparing area under the curve (AUC) between the 4 models. The AUC in model 1ofclinical characteristics only, including age, sex, smoking, alcohol drinking, and tumor site, was 0.590 (95% CI = 0.527 to 0.653), which increased to 0.669 (95% CI = 0.609 to 0.730) in model 2, after addition of the KDM4A expression levels. Furthermore, the AUC of clinical factors plus pathological characteristics was 0.695 (95% CI = 0.637 to 0.753) in model 3, which increased to 0.733 (95% CI = 0.678 to 0.789) in model 4. When KDM4A was added to conventional risk factors, the AUC reached the highest level compared with all the other models. Thus, the results indicated that KDM4A showed excellent discrimination and might be a better predictor of OSCC clinical outcome compared to the clinicopathological risk factors. ROC curves constructed for the models 3 and 4 (without and with KDM4A) are shown in Figure 4.

## DISCUSSION

In this study, we showed that increased KDM4A expression is an excellent predictor of poor clinical outcome in OSCC patients. The prognostic value of this epigenetic marker was verified in the multicenter cohort of 313 patients, and confirmed by external validation cohort of patients on TCGA dataset. KDM4A expression has considerable potential to predict prognosis of patients with OSCC.

The precise mechanism by which KDM4A expression increases in OSCC, producing bad outcomes, is unknown. Epigenetic factors mediate interactions between the environment and genome [6]. Lysine methylation is one of the most common histone posttranslational modification that affects chromatin structure [14, 15]. Dysregulation of histone lysine methyltransferases and demethylases has been found to be involved in tumor development and progression [5, 16]. KDM4A is a transcriptional cofactor and demethylase that catalyzes demethylation of lysines 9 and 36 on histone H3. Various studies have shown that KDM4A was overexpressed in breast, colorectal, lung, prostate, and other tumors [5, 17]. Recently, KDM4A has been identified as an important epigenetic factor that promotes HNSCC invasion and metastasis by stimulating AP-1 expression. The histone demethylase activity of KDM4A was essential for both AP-1 recruitment and its feedback activation loop [7]. However, the impact of KDM4A on prognosis in OSCC patients has not yet been reported. Due to the osculation of oral cavity and external environment, our study could provide new insights into the OSCC research field.

TNM stage is a well-known, useful index for OSCC prognosis in clinical practice, but the current TNM staging system has critical limitations in predicting the survival of patients with OSCC. TNM stage is always used to predict the process and assess the risk for patients with OSCC, and the patients with same TNM stage usually have various survival time [18–20]. The current TNM lacks reproducibility and clinical relevance, and also overlooks the site-specific and depth of invasion. Moreover, many OSCC are not detected easily. Thus, molecular markers are needed to assist doctors in clinical practice and achieve more applicable systems in the future [21, 22]. Through an analysis of tumor tissues from three cohorts, we found that KDM4A was differently expressed in OSCC tissues with different survival time. Moreover, according to clinical pathological indicators, the increased expression of KDM4A in OSCC was significantly associated with advanced TNM stage, indicating that KDM4A may have critical roles in the prognosis of OSCC patients. In this study, the high expression of KDM4A was found to be an independent risk factor for mortality risk of OSCC with a dose-response trend. This is a significant result because current diagnostic procedures cannot distinguish between clinically aggressive and clinically indolent OSCC, resulting in more occurrence of excessive medical treatment.

We developed prediction models to provide an efficient tool to identify and quantify poor prognosis in the OSCC patients. The HR for strong KDM4A positivity was 2.426 in model 2, but reduced to 2.191 in model 4 when pathological features were considered (Table 3). Even though, the pathological features had an impact on the predictive ability of KDM4A, KDM4A remained a major statistically significant predictor, even in the presence of the pathological status. Specifically, KDM4A and pathological characteristics together showed AUC exceeding 0.733. In general, the KDM4A expression level can classify patients with the same TNM stage into high- and low-risk groups with significantly different survival prospects, indicating that KDM4A can improve the accuracy of survival prediction. This finding might assist doctors to select high-risk patients for adjuvant therapy in addition to traditional surgery, which may lead to more efficient treatment options, as well as closer follow-up.

Our model identified two risk factors from health history, smoking and drinking, but both of them demonstrated no biologically relevant and statistically significant risks. Several studies have reported that leukoplakia on the tongue or the floor of the mouth showed a high risk of malignant transformation, while other studies have found no oral subsites at high risk [23]. However, no other literature examined the impacts of the tumor site on patient outcome. We found that tumors on the floor of the mouth, lips, or palate were statistically significant risk factors for survival time. Therefore, consideration of the tumor site would add important value to the risk prediction process.

The advantage of our study is that it was prospective, with all the specimens collected from individuals who underwent oral cancer operation. Moreover, the study outcome was assessed by regular follow-ups. Acknowledged predictors of oral cancer, including clinicopathological indicators, were added into the models to accurately examine the incremental value of KDM4A.

This study, however, had several limitations, including that the participants were treated surgically and some of them abandoned treatment or selected chemoradiotherapy; hence, this may limit the generalizability of our findings. For this reason, the ability of KDM4A to predict death risk of OSCC needs to be further validated in a larger study population. Moreover, only postoperative survival time was used for the model, while tumor recurrence was not considered. Even though the predictive power of KDM4A was reinforced in this study, the recurrence data should be integrated into our prediction model.

In conclusion, we showed that KDM4A is significantly and independently associated with the clinical outcome of OSCC, and provides a new option to the established prognostic factors. Further validation studies in prospective cohorts from different institutions are needed to test the prognostic power of KDM4A before it is applied clinically.

## MATERIALS AND METHODS

### Study population and data collection

All research designs had Institutional Review Board approval, and all subjects provided a voluntary informed consent to participate in the study. A total of 313 patients were enrolled. Patients were surgically treated at three stomatological centers, the West China Hospital of Stomatology, Sichuan University (Chengdu, China), the Guangdong Provincial Stomatological Hospital& the Affiliated Stomatological Hospital of Southern Medical University (Guangzhou, China), and the Chinese PLA (People's Liberation Army) General Hospital (Beijing, China); surgical margins were more than 5 mm. Tumor diagnosis was performed by senior specialists in oral surgery and expert pathologists.

Clinicopathological data were collected through assessment of medical records of OSCC patients treated surgically at each center, including gender, age, smoking and alcohol consumption history, tumor site and size, cell differentiation, lymph node metastasis, and clinical TNM stage. Patients were eligible for data collection if they were histologically diagnosed with OSCC and previously treated with primary surgery. The exclusion criteria were as follows: (1) recurrent or metastatic disease, (2) malignant disease of salivary glands, tonsils, and those located in oropharynx or hypopharynx, and (3) patients who had received prior or postoperative chemotherapy. Telephone interview was conducted routinely, at least for 6 months during the follow-up. Survival time was defined as the time from diagnosis to death or the last follow-up visit, and was checked at the last telephone interview in May 2014, irrespective of whether the patient was alive or not.

An independent cohort of 107 patient specimens with head and neck SCC (HNSCC), obtained from The Cancer Genome Atlas (TCGA) database (2015, http://tcga-data.nci.nih.gov/tcga/), was used as the external cohort to validate the prognostic value of KDM4A. The distribution of KDM4A mRNA expression in TCGA database (the base 2 logarithm of RPKM, Reads Per Kilobase of exon model per Million mapped reads) is closed to normal distribution and the median was chose to be the cutoff.

### TMA design and immunohistochemistry

Tissue cores of OSCC from 313 patients were used to construct TMAs using the Tissue Arrayer device (Beecher Instrument, MD, USA). All sections were histologically reviewed, and the representative areas of different clinical and pathological stages were extracted and arrayed. Thus, three different TMA slides were constructed from formalin-fixed, paraffin-embedded blocks of 313 OSCCs from three medical centers.

TMA slides were heated at 56°C for 2 hours. Then, they were deparaffinized twice in xyleneand rehydrated in a series of graded alcohols. Antigen retrieval was performed by pressure cooking (Decloaking chamber, Biocare Medical, Pacheco, CA, USA) in 0.01M citrate buffer (pH 6.0) at 120°C for 20 minutes. Endogenous peroxidase activity was blocked with 3% hydrogen peroxide solution for 15 minutes, followed by blocking non-specific binding with 1% (w/v) bovine serum albumen in PBS for 1 hour. Tissue sections were incubated with the primary antibody rabbit polyclonal KDM4A (Bethyl, Montgomery, TX, USA), diluted in 50mM Tris-HCl (1:50, pH 7.6), 150mM NaCl, and 0.1% Tween20 at 4°C overnight, followed by incubations with the ChemMate EnVision/HRP, Rabbit/Mouse (ENV) reagent of Envision Detection Kit (Dako Corporation, Carpinteria, CA, USA) for 60 minutes. The immunocomplexes were visualized using the ChemMate DAB+ chromogen of Envision Detection Kit, and counterstained with Harris hematoxylin.

### Semi-quantitative analysis

The degree of immunostaining, which is proportional to KDM4A expression, was evaluated blindly by two pathologists, based on the intensity of the staining and the percentage of positive tumor cells. The intensity of staining was scored as follows: 0, no color; 1, light yellow; 2, light brown; 3, brown. The number of positive cells was scored as follows: 0, < 5%; 1, 5–25%; 2, 25–50%; 3, > 50%. The two grades were multiplied together, producing scores from 0 to 9 that were classified as follows: weak staining (0–4 scores); strong staining (6–9 scores). Importantly, all assays were stained at the same time, using the same reagents. All assays demonstrating inferior reactivity on positive controls were examined and repeated.

### Statistical analysis

Association between clinicopathological characteristics of the patients and KDM4A expression was analyzed with the chi-square and Kruskal-Wallis test for categorical and continuous variables, respectively. Sex, smoking, drinking, tumor site (buccal mucosa, tongue, gingiva and other sites including floor of the mouth, lips, and palate) and lymph node metastasis were classified as categorical variables; other variables were continuous variables.

Univariate logistic regression was used to assess the contribution of KDM4A expression, sex, age (< 60 or > 60), smoking, drinking, tumor site, cell differentiation, tumor size (small, T1/T2; large, T3/T4), lymph node metastasis, and clinical TNM stage (early stage, I/II; late stage, III/IV) in overall survival. Survival curves were determined with the Kaplan-Meier method, and the differences between the variables categories were compared using the log-rank test. The multivariate Cox proportional-hazards model was applied to evaluate risk predictors that were significantly associated with poor prognosis. Specifically, 4 models were developed: model 1, clinical characteristics; model 2, clinical characteristics and KDM4A expression; model 3, clinical and pathological characteristics; model 4, clinicopathological characteristics and KDM4A expression.

To evaluate discriminatory accuracy of the models at the individual level of prognosis, the sensitivity and specificity of the given data were identified, and the area under the receiver operating characteristic (ROC) curve of each model was determined.

All statistical analyses were performed using SPSS (Statistical Package for the Social Sciences) v.17.0 software (SPSS Inc.). For unadjusted comparisons, *P* < .05 was considered statistically significant.
